# Target volumes comparison between postoperative simulation magnetic resonance imaging and preoperative diagnostic magnetic resonance imaging for prone breast radiotherapy after breast‐conserving surgery

**DOI:** 10.1002/cam4.6956

**Published:** 2024-01-22

**Authors:** Ying Jin, Changhui Zhao, Lizhen Wang, Ya Su, Dongping Shang, Fengxiang Li, Jinzhi Wang, Xijun Liu, Jianbin Li, Wei Wang

**Affiliations:** ^1^ Department of Radiation Oncology, Shandong Cancer Hospital and Institute Shandong First Medical University and Shandong Academy of Medical Sciences Jinan China; ^2^ Department of Oncology, Jinan Third People's Hospital Jinan Cancer Hospital Jinan China; ^3^ Department of Medical Physics, Shandong Cancer Hospital and Institute Shandong First Medical University and Shandong Academy of Medical Sciences Jinan China

**Keywords:** breast cancer, deformable image registration, preoperative diagnostic magnetic resonance image, target volume comparison

## Abstract

**Background:**

This study investigated the differences in target volumes between preoperative magnetic resonance imaging (MRIpre) and postoperative MRI (MRIpost) for breast radiotherapy after breast‐conserving surgery (BCS) using deformable image registration (DIR).

**Methods and Materials:**

Seventeen eligible patients who underwent whole‐breast irradiation in the prone position after BCS were enrolled. On MRIpre, the gross tumor volume (GTV) was delineated as GTVpre, which was then expanded by 10 mm to represent the preoperative lumpectomy cavity (LC), denoted as LCpre. The LC was expanded to the clinical target volume (CTV) and planning target volume (PTV) on the MRIpre and MRIpost, denoted as CTVpre, CTVpost, PTVpre, and PTVpost, respectively. The MIM software system was used to register the MRIpre and MRIpost using DIR. Differences were evaluated regarding target volume, distance between the centers of mass (dCOM), conformity index (CI), and degree of inclusion (DI). The relationship between CI_LC_/CI_PTV_ and the clinical factors was also assessed.

**Results:**

Significant differences were observed in LC and PTV volumes between MRIpre and MRIpost (*p* < 0.0001). LCpre was 0.85 cm^3^ larger than LCpost, while PTVpre was 29.38 cm^3^ smaller than PTVpost. The dCOM between LCpre and LCpost was 1.371 cm, while that between PTVpre and PTVpost reduced to 1.348 cm. There were statistically significant increases in CI and DI for LCpost–LCpre and PTVpost–PTVpre (CI = 0.221, 0.470; DI = 0.472, 0.635). No obvious linear correlations (*p* > 0.05) were found between CI and GTV, primary tumor volume‐to‐breast volume ratio, distance from the primary tumor to the nipple and chest wall, and body mass index.

**Conclusions:**

Despite using DIR technology, the spatial correspondence of target volumes between MRIpre and MRIpost was suboptimal. Therefore, relying solely on preoperative diagnostic MRI with DIR for postoperative LC delineation is not recommended.

## 1 INTRODUCTION

Breast‐conserving surgery (BCS) is an established treatment for early‐stage breast cancer.^1^ When combined with postoperative radiotherapy, it significantly reduces the risk of postoperative recurrence and mortality.^2^, ^3^ Local recurrence occurs predominantly near the lumpectomy cavity (LC) after BCS, accounting for 65% to 85% of cases.^2^, ^4^ Therefore, accurate identification and delineation of the LC after BCS is crucial. The gold standard for LC delineation involves postoperative computed tomography (CT) imaging, which considers seroma, surgery clips, and glandular tissue rupture.^5^, ^6^ Several factors influence LC identification, including suturing technique, cavity visualization score (CVS), number of surgery clips, the interval between BCS and CT simulation scan, postsurgical breast distortion, and the radiation oncologist's subjective perception and experience.^7^, ^8^, ^9^, ^10^ Consequently, relying solely on localized CT images causes significant inconsistencies in LC delineation.

Compared with CT, magnetic resonance imaging (MRI) provides superior soft tissue contrast. The LC target volume delineated based on the MRI localization image is comparable to that based on CT localization, whether in the supine or prone position.^11^ Moreover, MRI offers easier LC delineation.^12^, ^13^ However, the visibility of the seroma also influences LC determination on MRI.^14^ Consequently, numerous studies have investigated the feasibility of co‐registering CT and MRI to guide LC delineation.^11^, ^15^, ^16^ Nevertheless, LC delineation, including seroma, through CT‐MR matching fails to reduce inter‐subject variability.^15^ In our previous study, we observed significant advantages in delineating the LC using delayed‐enhancement T1‐weighted magnetic resonance (DE‐T1WI‐MR) images acquired 10 min after injection, even for patients without a seroma or with a poor CVS (≤2).^17^

Reviewing preoperative images and considering the primary tumor location, size, and adjacent relationship can reduce interobserver variability in LC delineation. However, no studies have investigated the correlation between the LC on simulated MRI and the preoperative primary tumor on diagnostic MRI in the prone position. Therefore, our primary objective was to evaluate the differences in target volume between preoperative and simulated MRI scans in the prone position before BCS, to assess the rationality and feasibility of preoperative MRI‐guided LC delineation. Our secondary objective was to analyze the correlation between the target volume matching index and the clinical and anatomical factors to identify patients suitable for preoperative MRI‐guided LC delineation.

## 2 METHODS

### 2.1 Patient selection

This study enrolled patients with negative margins who underwent BCS and irradiation in the prone position. All the patients were newly diagnosed with histologically confirmed invasive breast carcinoma. The inclusion criteria were as follows: early‐stage breast cancer (pT1‐2, N0, and M0) and the presence of at least five titanium surgical clips placed in the LC. Patients were treated with simultaneous integrated boost intensity‐modulated radiation therapy (SIB‐IMRT) or sequential boost‐IMRT after whole breast irradiation. Before surgery, all patients underwent preoperative diagnostic MRI and postoperative prone CT simulations. This was followed by delayed‐enhancement magnetic resonance imaging with 10th‐minute DE‐T1WI scans in the prone position.

The exclusion criteria were neoadjuvant chemotherapy/immunotherapy, breast reconstruction, multifocal or multicentric disease, chronic lung disease, prior thoracic radiotherapy, and limited shoulder joint mobility. This study was approved by the Research Ethics Committee of Shandong Cancer Hospital and the Institute Ethics Committee (SDTHEC201703014). The requirement for written informed consent from patients was waived because this is a retrospective single‐institution cohort study.

### 2.2 Image acquisition

All patients underwent three‐dimensional (3D) CT simulation scans followed by MR simulation scans while breathing freely. Patients were positioned prone on a dedicated prone breast bracket (CIVCO Horizon™ Prone Breast Bracket‐MTHPBB01) with their heads facing downward on the dedicated headrest and both hands naturally raised to hold the handle. The position of the board on the healthy side was adjusted to prevent the healthy breast from exerting tension on the affected side, allowing the affected breast to hang naturally in the treatment hole. Laser lights were collimated and marked on the affected breast, bilateral posterior axillary line, and back. A large‐aperture CT simulator (Philips Medical Systems, Inc., Cleveland, OH, USA) was used to scan the patients, covering a range from the cricothyroid membrane to the lower border of the liver.

Prone MRI examinations were conducted using a 3.0 T, 70‐cm bore MR positioning machine (750 W, General Electric Co., Boston, USA) from GE in the United States. The same breast bracket and scanning position as the CT simulation positioning scanning were used. The imaging protocol consisted of acquiring T2‐weighted images with fat suppression to minimize motion artifacts in the resting state. Subsequently, in a breath‐holding state, DE‐T1WI with fat suppression was performed 10 min after contrast administration. For the enhanced sequences, 15 mL of gadopentetate dimeglumine was administered intravenously at a rate of 2 mL/s. Subsequently, 20 mL of normal saline was injected to ensure complete absorption of the contrast medium into the body.

Both MR and CT images were acquired with a slice thickness of 3 mm. Subsequently, the MRI and CT datasets were exported to the MIM version 6.8.3 software (Cleveland, USA) for registration and delineation.

### 2.3 Target volume delineation

**FIGURE 1:**
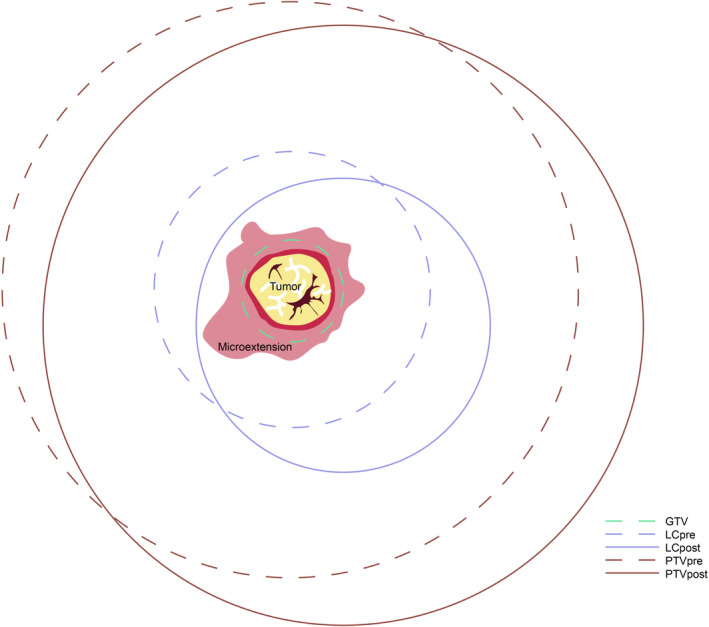
The simulation schematic of target volumes.

**FIGURE 2:**
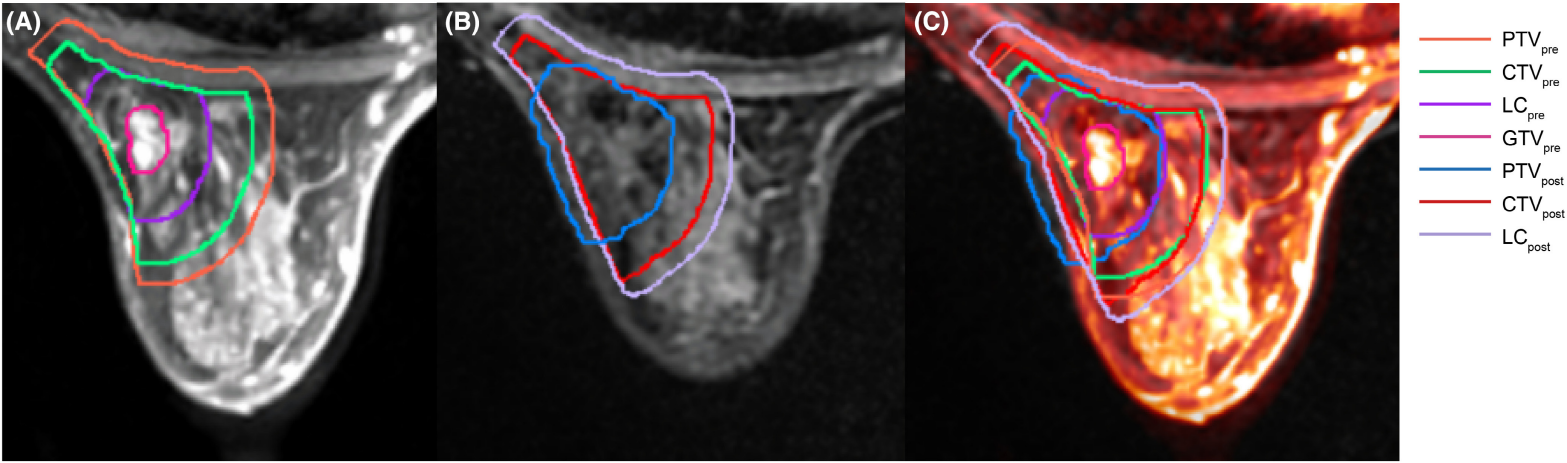
Target volumes delineation based on preoperative prone diagnostic MRI (A), postoperative prone simulation MRI (B), and both of them based on DIR (C).

The hyperintense T1WI area (excluding the hyperintense glandular tissue surrounding the primary tumor) was used as a guide for delineating the gross tumor volume (GTV)pre on preoperative MRI. The LCpre was generated by expanding the GTVpre by 10 mm while ensuring a 5 mm distance from the skin surface and avoiding extension into the pectoralis muscles and/or muscles of the chest wall (Figure 2A). In contrast, the LCpost was delineated based on CT and MRI scans, incorporating the presence of seroma, surgical clips, and noticeable variations in the glandular breast tissue (Figure 2B). The specific delineations of target volumes for clinical target volume (CTV) and planning target volume (PTV) are presented in Table 1. The simulation diagram of the target volume is illustrated in Figure 1.

**TABLE 1:** Target volumes delineation for breast cancer before and after BCS.

Target volumes		Delineation
GTV	GTVpre	Primary tumor delineated on postcontrast sagittal fat‐suppressed T1‐weighted MRI
LC	LCpre	GTVpre with a 10 mm expansion, and trimmed 5 mm from skin surface and not extend into the pectoralis muscles and/or muscles of the chest wall
	LCpost	Seroma, surgical clips, and notable differences in the glandular breast tissue all included based on CT and MRI
CTV	CTVpre	LCpre with a 10 mm expansion, and trimmed 5 mm from skin surface and not extend into the pectoralis muscles and/or muscles of the chest wall
	CTVpost	LCpost with a 10 mm expansion, and trimmed 5 mm from skin surface and not extend into the pectoralis muscles and/or muscles of the chest wall
PTV	PTVpre	CTVpre expanded with a 5 mm margin based on setup error and trimmed 5 mm from skin surface
	PTVpost	CTVpost expanded with a 5 mm margin based on setup error and trimmed 5 mm from skin surface

Abbreviations: CTV, clinical target volume; GTV, gross tumor volume; LC, lumpectomy cavity; PTV, planning target volume.

### 2.4 Deformable image registration

In this study, all images were subjected to deformable image registration (DIR) using the MIM system. The automatic DIR process involved an initial rigid image registration (RIR), followed by manual adjustments to refine the local alignments and subsequent RIR. During the initial registration, the postoperative MRI served as the primary sequence, whereas the postoperative CT image served as the secondary sequence. After completion of the automatic RIR, further evaluation and refinement of the registration were performed using the Reg Reveal and Reg Refine tools. Key anatomical landmarks such as the nipple, skin, ribs, and sternum were used to guide the registration process. In the second registration step, delineated postoperative MRI was used as the primary sequence, and preoperative MRI was used as the secondary sequence. The adjusted image was then subjected to another round of DIR (Figure 2C).

### 2.5 Volume comparison

**FIGURE 3:**
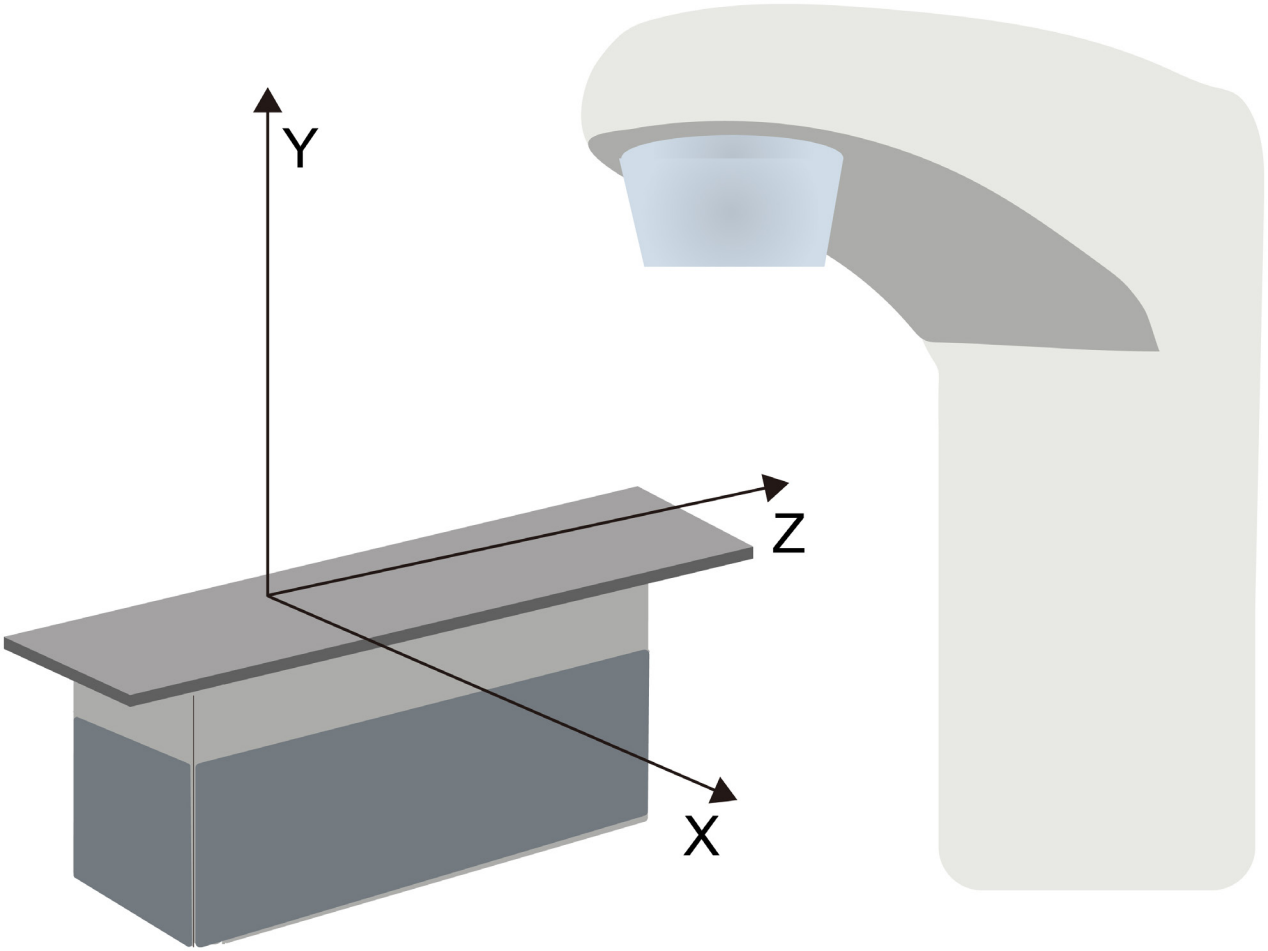
Corresponding 3D space coordinates [lateral (*x*), anteroposterior (*y*), and superoinferior (*z*)].

After completing the DIR process using the MIM software, the volumes of the target areas and their corresponding 3D space coordinates [lateral (*x*), anteroposterior (*y*), and superoinferior (*z*)] were obtained (Figure 3). The differences in coordinate values between the centroids of the two target areas were denoted as Δ*x*, Δ*y*, and Δ*z*, respectively. Additionally, the distance between the centers of mass (COM) was calculated using the following formula:

$$\text{∆}V={\left(\text{∆}x^{2}+\text{∆}y^{2}+\text{∆}z^{2}\right)}^{1/2}$$

A Δ*V* value of 0 indicates that the centers of the target areas are in the same position.

The conformity index (CI) and degree of inclusion (DI) were compared for LCpre and LCpost, and for PTVpre and PTVpost. A CI or DI value of 1 indicated complete unity between the volumes. The CI, which measures the overlap between volumes A and B, was calculated using the following formula:

$$\mathrm{CI}\left(A,B\right)=\frac{A\cap B}{A\cup B}$$

Similarly, the DI, which represents the percentage overlap between volumes *A* and *B* relative to volume *A*, was defined as:

$$\mathrm{DI}\left(A,B\right)=\frac{A\cap B}{A}$$

### 2.6 Clinical data collection

**FIGURE 4:**
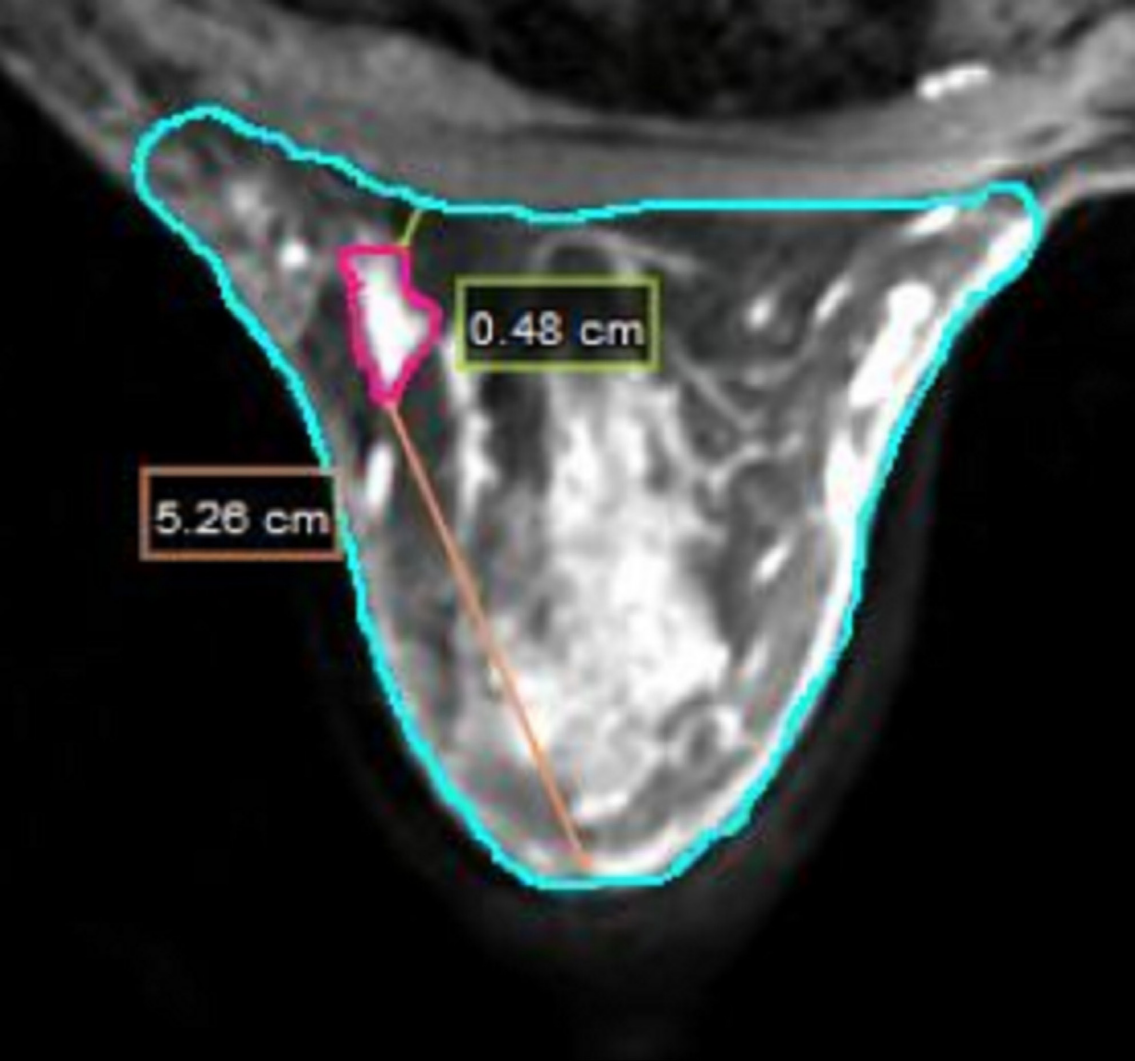
The distance measurement from GTV to nipple and chest wall.

Various parameters were collected for analysis, including body mass index (BMI), tumor location, CVS, primary tumor size and volume, ratio of primary tumor volume to breast volume, and distance from the primary tumor to the nipple and chest wall (Figure 4). BMI was calculated as weight divided by height squared (BMI = $\frac{\text{weight}}{{\text{height}}^{2}}$). The CVS scores range from 1 to 5, representing increasingly distinct margins and a more homogeneous appearance.^18^

### 2.7 Statistical analysis

The data were analyzed using SPSS software (version 26.0; IBM Corporation, Armonk, NY, USA). Non‐normally distributed data are described using medians and ranges. The Wilcoxon signed‐rank test was used to compare the target volumes and related parameters. Additionally, Spearman's rank correlation analysis was used to assess the correlations between the spatial indices and clinical data.

## 3 RESULTS

### 3.1 Patient characteristics

Ninety‐seven patients with negative margins who underwent prone irradiation after BCS from September 2018 to December 2022 were initially enrolled in this study. Among them, 41 patients were excluded owing to a lack of preoperative MRI records at our hospital, and an additional 21 patients were excluded owing to a lack of postoperative MR localization. Overall, 17 patients met the inclusion criteria and were included in the final analysis. The median age of the cohort was 44 years (range: 33–62 years). All patients had T1‐T2 N0M0 stage breast cancer, with invasive ductal carcinoma being the most prevalent histological subtype, accounting for 88.24% of all cases. Lumpectomy was the chosen surgical approach for all patients, resulting in the successful attainment of negative tumor margins in a single procedure. The median CVS score was 2. The detailed cohort data are presented in Table 2.

**TABLE 2:** Characteristics of 17 patients studied.

Characteristics	No. of cases (%)
Age (years), median (range)	44 (33–62)
Breast side	
Left	10 (58.82)
Right	7 (41.18)
Tumor location	
OUQ	9 (52.94)
OLQ	2 (11.76)
IUQ	6 (35.29)
ILQ	0
Central portion of breast	0
Tumor characteristics	
Invasive ductal carcinoma	15 (88.24)
Invasive lobular carcinoma	1 (5.88)
Mixed mucinous carcinoma	1 (5.88)
CVS	
1	7 (41.18)
2	2 (11.76)
3	4 (23.53)
4	1 (5.88)
5	3 (17.65)
Tumor diameter	
≥10 mm <20 mm	10 (58.82)
≥20 mm <30 mm	7 (41.18)

Abbreviations: CVS, cavity visualization score; ILQ, inter lower quadrant; IUQ, inter upper quadrant; OLQ, outer lower quadrant; OUQ, outer upper quadrant.

### 3.2 Target volume evaluation

**FIGURE 5:**
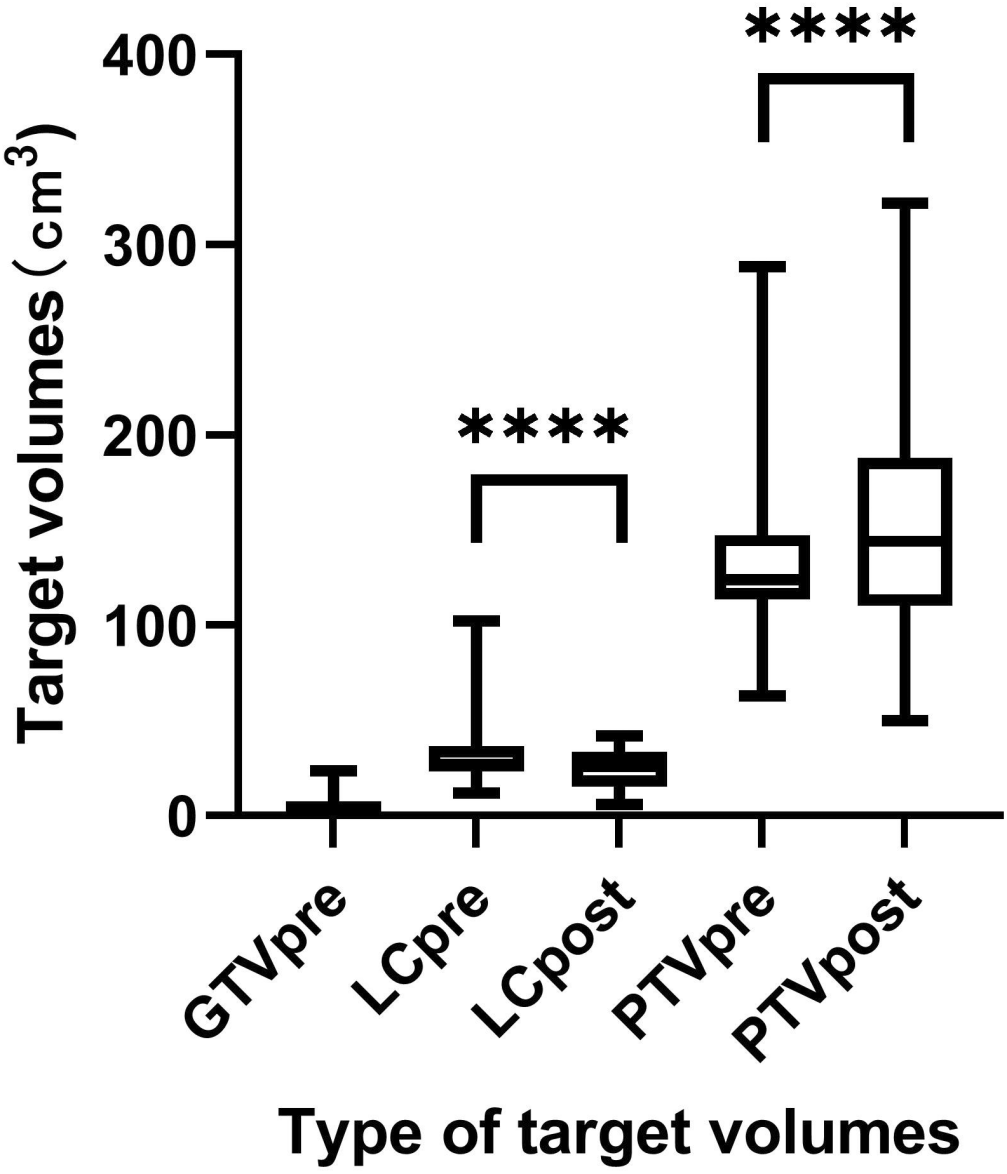
Target volume defined using preoperative prone MRI and postoperative prone MRI (cm^3^). **** *p* ＜0.0001

The median volume of the primary tumor was 3.05 cm^3^. The comparison of the target volumes between pre‐ and postoperative MRI based on DIR for LC and PTV is illustrated in Figure 5. The median volume of LCpost was 0.85 cm^3^ larger than LCpre. There was a statistically significant difference between PTVpre and PTVpost, with volumes of 114.69 and 144.07 cm^3^, respectively. No significant correlation was observed between LCpre and LCpost (*r* = 0.321, *p* = 0.209). Similarly, there was also no significant correlation between PTVpre and PTVpost (*r* = 0.424, *p* = 0.091).

The displacements between LCpre and LCpost were 0.5, 0.71, and 0.7 cm in the *x*, *y*, and *z* directions, respectively. The dCOM was 1.371 cm (range: 0.533–5.447). The 3D motion amplitude of the LC centroid caused by surgery and delineation showed a significant difference only in the *z*‐direction in this cohort (*t* = −3.280, *p* = 0.005). After expanding with a 15 mm margin and trimming 5 mm from the skin surface, the centroid shifts between PTVpre and PTVpost in the *x*, *y*, and *z* directions were 0.35, 0.67, and 0.61 cm, respectively. The dCOM was reduced to 1.348 cm (range: 0.406–3.642 cm) (Table 3). The CI and DI between LCpre and LCpost were 0.221 (range: 0.041–0.516) and 0.472 (range: 0.108–0.804), respectively. Compared with LC, the CI and DI between PTVpre and PTVpost increased to 0.470 (range: 0.245–0.721) and 0.635 (range: 0.347–0.823), respectively.

**TABLE 3:** Evaluation of target volume parameters using preoperative and postoperative prone MRI with DIR.

Parameters	Median	Range
dCOM		
LCpre–LCpost	1.371	0.533–5.447
PTVpre–PTVpost	1.348	0.406–3.642
CI		
LCpre–LCpost	0.221	0.041–0.516
PTVpre–PTVpost	0.470	0.245–0.721
DI		
LCpre–LCpost	0.472	0.108–0.804
PTVpre–PTVpost	0.635	0.347–0.823

Abbreviations: CI, the conformity index; dCOM, the distance between the COM of the targets; DI, the degree of inclusion.

### 3.3 Correlation analysis

No significant linear correlations were observed between the CI and the GTV volume, the ratio of the primary tumor volume to the breast volume (R_GTV/breast_), the distance from the primary tumor to the nipple (d_GTV‐nipple_), chest wall (d_GTV‐CW_), CVS, and BMI (Table 4).

**TABLE 4:** The correlation between the CI and clinical/anatomic factors.

Parameters	GTV	R_GTV/breast_	d_GTV‐nipple_	d_GTV‐CW_	BMI	CVS
CI (LCpre–LCpost)						
*r*‐value	−0.090	0.021	0.206	−0.277	−0.450	0.276
*p*‐value	0.947	0.937	0.428	0.282	0.863	0.284
CI (PTVpre–PTVpost)						
*r*‐value	−0.175	0.045	0.062	−0.230	−0.196	0.046
*p*‐value	0.502	0.865	0.814	0.374	0.450	0.861

Abbreviations: d_GTV‐CW_, the distance from the primary tumor to the chest wall; d_GTV‐nipple_, the distance from the primary tumor to the nipple; R_GTV/breast_, the ratio of the primary tumor volume to the breast volume.

## 4 DISCUSSION

Postoperative radiotherapy after BCS is crucial for reducing the local and regional recurrence rate and improving long‐term survival outcomes.^4^, ^19^ Accurate determination of the target volume of the LC is essential for effective radiotherapy, whether in the supine or prone position. In addition to using surgical clips, seromas, and incisions to aid LC delineation, the guidance provided by preoperative imaging, particularly MRI, should not be overlooked. MRI offers superior soft tissue resolution compared with CT and facilitates better identification of the primary tumor.^20^, ^21^, ^22^ The selection of images for radiotherapy planning is an ever‐evolving process. Recent emphasis on the significance of preoperative MRI selection has been highlighted across various tumor radiotherapy contexts.^23^, ^24^ Prone positioning during whole‐breast radiotherapy offers advantages such as improved target conformity, dose homogeneity, reduced dose to organs at risk, and mitigation of acute radiation skin injury,^25^, ^26^ especially in patients with larger breasts.^27^, ^28^, ^29^, ^30^ Consequently, radiotherapy in the prone position after BCS is increasingly being used in patients with early‐stage breast cancer. This study aimed to investigate the use of preoperative MRI in guiding LC delineation in patients with BCS.

Preoperative imaging plays a vital role in providing valuable locational information for breast carcinomas. Van der Leij et al.^31^ confirmed that the postoperative tumor bed, based on CT images in the supine position, was significantly larger than the preoperative GTV by 7.71 m^3^. Combining CT simulation scanning with MRI improved soft tissue contrast for target volume delineation in adjuvant radiotherapy for patients with breast cancer with a high CTV.^14^, ^32^ In this study, we aimed to evaluate the use of preoperative MRI (pre‐MRI) in guiding LC delineation, as defined by postoperative CT (post‐CT) in the prone position. Previous studies by Yu et al.^33^ and Dong et al.^10^ demonstrated that CTVpreMR was significantly smaller than CTVpostCT. To minimize the differences between the scan images, we compared the target volumes delineated using postoperative simulation MRI and preoperative diagnostic MRI. LCpre was smaller than LCpost; however, the difference was only 0.85 cm^3^, which is smaller than that in previous studies.^10^ This difference may be attributed to our delineation based on 10 min post‐injection with delayed‐enhancement T1‐weighted MR images.^17^ Considering the intrafractional and setup errors, an applied margin of 10 mm is commonly used for external‐beam partial breast irradiation. In our study, we expanded a 0.5 cm margin around CTV using SIB technology to obtain the PTV. We observed that the median PTVpre was 29.38 cm^3^ smaller than the median PTVpost (*p* < 0.0001). Consequently, preoperative MRI can contribute to reducing target volume, which may mitigate acute skin toxicity. This is particularly relevant as breast volume is a known risk factor for radiation dermatitis and associated symptoms, among other contributing factors.^34^ Therefore, preoperative MRI may help reduce the target volume; however, its value in guiding postoperative LC delineation requires further investigation.

We further investigated the correlation and spatial alignment between the LC and PTV obtained from pre‐ and postoperative MRI scans using DIR. DIR offers improved GTV registration overlap compared with RIR.^35^ During the RIR process, the maximal overlap of glandular tissue was achieved based on the tumor location and anatomical landmarks. All images were acquired in the prone position to enhance consistency and homogeneity. However, the correlation between LC and PTV was relatively weak (*r* = 0.321, *p* = 0.209 vs. *r* = 0.424, *p* = 0.091). Even when using MRI, there can be varying degrees of underestimation and overestimation of the tumor size.^36^, ^37^, ^38^ The correlation coefficient between visible lesions on MRI and primary tumor volume was 0.76, with an average difference of 1.3 mm between the two largest diameters.^39^ However, the spatial matching index between postoperative simulation MRI and preoperative diagnostic MRI was suboptimal, with the CI and DI below 0.75. The centroid shifts between LCpre and LCpost showed significant differences only in the *z*‐direction, which may be attributed to changes in breast gravity after BCS. During the DIR process, we observed that the two patients with the best breast‐space matching between the preoperative diagnostic MRI and postoperative MRI had primary tumors located closer to the chest wall than the median distance. Considering the different boundary restrictions during postoperative target area expansion (LC to CTV) for tumors near the chest wall and skin and uncertainties regarding anatomical differences (R_GTV/breast_, d_GTV‐nipple_, d_GTV‐CW_, CVS, and BMI), we investigated the potential relationship between various clinical and anatomical parameters. However, no statistically significant correlations were observed between these parameters.

Several factors contribute to the inadequate spatial matching of the breasts. The primary factor is the variation in the treatment boards used for preoperative and postoperative MRI. The diagnostic MRI board allows both breasts to hang freely, whereas the postoperative localization MRI board restricts the contralateral breast from the same side, allowing only the treated breast to hang freely. Moreover, most surgeons subjectively perform BCS based on palpable boundaries, resulting in asymmetric excision of the primary tumor. The excised specimen and delineated LC do not correlate with the visible tumor volume on preoperative MRI.^40^, ^41^ Additionally, seromas can undergo significant volume and shape changes in the immediate postoperative period,^42^, ^43^, ^44^ gradually decreasing or even disappearing over time.^45^

However, this study has some limitations. First, the sample size was relatively small. Second, there may be constraints associated with the DIR technique. DIR can capture non‐rigid deformations of the breast; however, the intricate tissue structure, volume reduction, and changes after breast resection can introduce inaccuracies in the matching process when using the DIR tool. The observed differences, with substantial potential clinical implications for local tumor control, necessitate further investigation in upcoming research trials. It is important to emphasize that these findings are relevant primarily to patients receiving radiotherapy in the prone position. Table 5 presents a comparison of preoperative and postoperative breast tumor bed contours based on characteristics from various studies.

**TABLE 5:** Characteristics of different other studies based on comparison of preoperative and postoperative breast tumor bed contours.

Authors	NO.	Image	Volume (cm^3^/cc)		Intra/inter observer variation			
			Pre	Post		CI	ΔV (cm)	GMI
Yu et al.^33^	18	Pre‐MR vs. post‐CT	CTVpreMR+10: 34.33 cm^3^	GTVpostCT: 17.22 cm^3^	Intra	0.31	0.14	_
				CTVpostCT+10: 53.46 cm^3^ (all *p* < 0.05)		0.38	0.21	
Dong et al.^10^	22		CTV: dyn‐eTHRIVE: 34.191 ± 12.171 cm^3^	CT: 37.350 ± 13.699 cm^3^ (*p* = 0.065)		0.721 ± 0.052 (*p* = 0.093)	_	0.159 ± 0.063
			PTV: dyn‐eTHRIVE: 165.873 ± 37.476 cm^3^	CT: 181.246 ± 42.576 cm^3^ (*p* < 0.001)		0.844 ± 0.043 (*p* = 0.349)		0.060 ± 0.034 (all *p* < 0.05)
van der Leij et al.^31^	24	Pre‐CT vs. post‐CT	GTV: 0.97 cc	TB: 8.68 cc (*p* < 0.001)	Inter	CTVpre: 0.78	_	_
			CTV‐pre: 36.8 cc	CTV‐post: 41.0 cc (*p* = 0.789)		CTV‐post: 0.38 (*p* < 0.001)		
Jiang et al.^46^	10	Pre‐MR vs. post‐CT	CTV: dyn‐eTHRIVE: 27.15 ± 11.33 cm^3^	CT: 33.1 + 14.09 cm^3^		CTV: dyn‐eTHRIVE: 0.69, CT: 0.37	_	_
			PTV: dyn‐eTHRIVE: 144.28 ± 41.83 cm^3^	CT: 194.51 + 53.11 cm^3^ (all *p* < 0.001)		PTV: dyn‐eTHRIVE: 0.84, CT: 0.65 (all *p* < 0.001)		

Abbreviations: CI, conformity index; CT, computed tomography; CTV, clinical target volume; dyn‐eTHRIVE, dynamic‐enhanced T1 high‐resolution isotropic volume excitation; GMI, geographical miss index; GTV, gross target volume; MR, magnetic resonance; post‐CT, postoperative computed tomography; post‐MR, postoperative magnetic resonance; pre‐CT, preoperative computed tomography; pre‐MR, preoperative magnetic resonance; PTV, planning target volume; TB, tumor bed; ΔV, the distance between the distance between the centers of mass of the target of the targets.

## 5 CONCLUSION

In this study, we investigated the feasibility of using DIR to enhance LC delineation by comparing preoperative diagnostic MRI with postoperative localized MRI. These findings suggest that achieving spatial consistency of the target volume between preoperative and postoperative MRI, particularly in prone breast irradiation, remains challenging even with DIR. Therefore, relying solely on preoperative diagnostic MRI based on DIR to guide postoperative LC delineation is not recommended. Additionally, preoperative MRI does not offer personalized guidance for LC delineation based on individual patient characteristics. However, this study provides valuable insights for optimizing LC delineation after BCS. Future research exploring image registration techniques based on machine learning and advanced algorithms has the potential to improve the spatial consistency between preoperative and postoperative MRI.

## AUTHOR CONTRIBUTIONS

**Ying Jin:** Data curation (lead); investigation (lead); methodology (lead); resources (lead); supervision (lead); validation (lead); visualization (lead); writing – original draft (lead). **Changhui Zhao:** Resources (supporting). **Lizhen Wang:** Resources (supporting). **Ya Su:** Resources (supporting). **Dongping Shang:** Resources (supporting). **Fengxiang Li:** Resources (supporting). **Jinzhi Wang:** Resources (supporting). **Xijun Liu:** Resources (supporting). **Jianbin Li:** Funding acquisition (supporting); methodology (supporting); resources (supporting); software (supporting); writing – review and editing (supporting). **Wei Wang:** Methodology (supporting); supervision (supporting); writing – review and editing (lead).

## FUNDING INFORMATION

This work was supported by the Taishan Scholars Program of Shandong Province (No. ts 20190982) and the National Natural Science Foundation of Shandong Provence (No. ZR2020QH260).

## CONFLICT OF INTEREST STATEMENT

The authors have no competing interests to declare relevant to this article's content.

## CONSENT TO PUBLISH

All authors provided consent to publish this study.

## Data Availability

The data that support the findings of this study are available from the corresponding author.
